# Predicting Molecular Weight Characteristics of Reductively Depolymerized Lignins by ATR-FTIR and Chemometrics

**DOI:** 10.1021/acssuschemeng.4c03100

**Published:** 2024-05-29

**Authors:** Luke A. Riddell, Peter de Peinder, Viviana Polizzi, Karolien Vanbroekhoven, Florian Meirer, Pieter C. A. Bruijnincx

**Affiliations:** † Faculty of Science, Organic Chemistry & Catalysis, Institute for Sustainable and Circular Chemistry, Utrecht University institution, Utrecht 3584CG, The Netherlands; ‡ Faculty of Science, Inorganic Chemistry & Catalysis, Institute for Sustainable and Circular Chemistry, Utrecht University, Utrecht 3584CG, The Netherlands; § VibSpec, Haaftenlaan 28, Tiel 4006 XL, The Netherlands; ∥ Sustainable Polymer Technologies team, Materials & Chemistry unit, Flemish Institute for Technological Research (VITO), Mol 2400, Belgium

**Keywords:** lignin, chemometrics, IR spectroscopy, PLS regression, molecular weight prediction

## Abstract

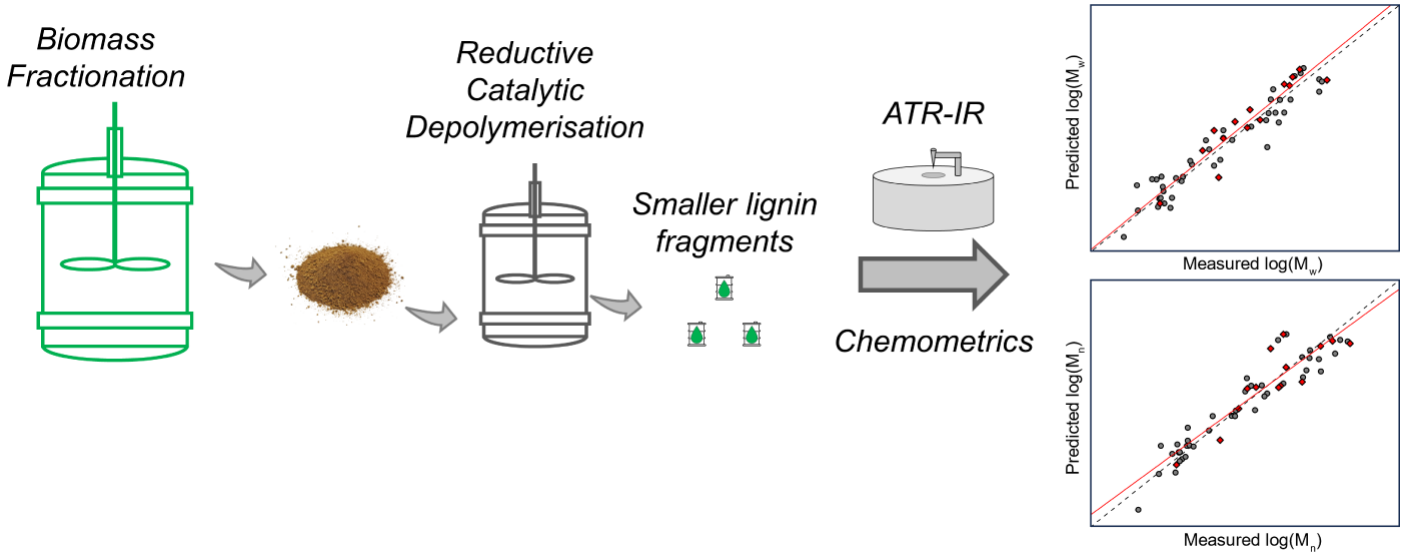

Recent scientific advances in the valorization of lignin, through e.g., (partial-)catalytic depolymerization, require equally state-of-the-art approaches for the analysis of the obtained depolymerized lignins (DLs) or lignin bio-oils. The use of chemometrics in combination with infrared (IR) spectroscopy is one avenue to provide rapid access to pertinent lignin parameters, such as molecular weight (MW) characteristics, which typically require analysis via time-consuming size-exclusion methods, or diffusion-ordered NMR spectroscopy. Importantly, MW serves as a marker for the degree of depolymerization (or recondensation) that the lignin has undergone, and thus probing this parameter is essential for the optimization of depolymerization conditions to achieve DLs with desired properties. Here, we show that our ATR-IR-based chemometrics approach used previously for technical lignin analysis can be extended to analyze these more processed, lignin-derived samples as well. Remarkably, also at this lower end of the MW scale, the use of partial least-squares (PLS) regression models well-predicted the MW parameters for a sample set of 57 depolymerized lignins, with relative errors of 9.9–11.2%. Furthermore, principal component analysis (PCA) showed good correspondence with features in the regression vectors for each of the biomass classes (hardwood, herbaceous/grass, and softwood) obtained from PLS-discriminant analysis (PLS-DA). Overall, we show that the IR spectra of DLs are still amenable to chemometric analysis and specifically to rapid, predictive characterization of their MW, circumventing the time-consuming, tedious, and not generally accessible methods typically employed.

## Abstract 2

The molecular weight of depolymerized lignins can be predicted using calibrated chemometric models built upon IR data. Further insights into important spectral predictors for this emerging class of lignin samples are presented.

## Introduction

As the second most abundant biopolymer on earth^1^ and the major renewable form of aromatic carbon,^2,3^ lignin is poised to be one of the key carbon feedstocks through the upcoming energy and materials transition. Its highly complex, variable, and heterogeneous composition is contingent on botanical origin, and further structural heterogeneity arises through variation in monolignol connectivity and functional group distribution within and between individual lignin chains as well as broad chain length dispersity (*Đ*). The inherent complexity of the lignin structure and often limited control over changes in the structure during processing continue to hamper valorization efforts. Indeed, these features directly contribute to the typically ill-defined physicochemical properties of isolated lignins and thus to “lack-of-fit” for many applications directly. Structurally, lignin is composed of syringyl (S), guaiacyl (G), and hydroxyphenyl (H) units, which form as a consequence of the radical polymerization of their phenylpropanoids (sinapyl, coniferyl, and *p*-coumaryl alcohols, respectively) within the plant cell wall.^4^ Hardwood lignins are typically rich in S and G rings, herbaceous/grass lignins contain a mixture of S, G, and H rings, and softwoods are typically purely G-based.^4,5^ Between the aromatic rings are a range of different linkages, with the most abundant being the β-O-4 ether linkage.^6^ Harvested biomass is typically subjected to harsh industrial pretreatment (e.g. kraft process) to delignify the valuable carbohydrate fractions. These conditions, however, rapidly cleave the (β-O-4) ether linkages within the lignin,^7^ with subsequent recondensation of the resulting fragments resulting in recalcitrant C–C bond formation.^8,9^ Additionally, as different conditions are employed in different biomass pretreatment methods (e.g. kraft, soda, and organosolv), different degradation pathways occur, meaning that each fractionation method leads to process-specific linkages and fragmentation patterns.^4^ These unintended modifications of the technical lignin structure further contribute to the structural heterogeneity, and the recalcitrant C–C linkages leave these lignins less suited for depolymerization strategies.^4,10,11^ Consequently, more C atom economic approaches for utilizing technical lignins hinge on the application of lignin fractionation,^12,13^ modification,^14^ or a combination of both^15,16^ to achieve suitably homogeneous samples for lignin-to-materials applications. While deep depolymerization of technical lignins may seem an attractive potential approach toward monoaromatics, its recalcitrance and complexity again hamper these efforts, limiting its efficiency and resulting in complex mixtures of products.^17^

Recently, strategies have been developed to preserve the β-O-4 linkages during delignification, operating under milder processing conditions, while still aiming for the maximum yield and purity of the carbohydrate fractions. Such so-called “lignin-first” strategies typically involve combined biomass fractionation, depolymerization, and stabilization, e.g., by hydrogenation of the reactive fragments prior to repolymerization, or preservation of β-O-4 bonds through classical acetal protection chemistry.^18−22^ Through the latter method, lignin maintains a β-O-4-rich structure, with minimal introduced C–C linkages, and is therefore much more amenable to controlled depolymerization after isolation. Both approaches thus provide access to a small handful of functionalized monophenolic compounds in high, almost theoretical yields, alongside some dimers and short-chain oligomers.^22^ Alternatively, mild organosolv pretreatment methods also aim at preservation of the β-O-4 linkages,^23−25^ some of which have already been demonstrated at the pilot scale.^26^ The isolated lignins are again relatively high in β-O-4 abundance and show fewer C–C interunit condensations than technical lignins isolated from more conventional processes. Additionally, mild organosolv lignins have been shown to be more amenable to deep depolymerization to monomers.^27^ Partial depolymerization of such lignins has also been reported, which aims to homogenize the lignin material by having the full sample converge on a material or oil of lower, tunable molecular weight (MW). These are primarily composed of oligomeric and/or short-chain polymeric lignin fragments^28−32^ with a smaller fraction of monophenolic components when compared to RCF processes.

For all of the processes above, MW is a key property, as both a proxy for the extent of depolymerization/recondensation and for the material properties and application potential of the products obtained. Indeed, it is a particularly dominant factor in material performance, influencing, for example, solubility,^12^ glass transition temperature (*T*_g_),^33,34^ and viscosity.^35^ Lignin(-oil) MW is typically quantified through size-exclusion or gel-permeation chromatography (SEC and GPC, respectively).^35−37^ These methods, however, are time-consuming, require tedious sample preparation and continuous (re)calibration of the system to ensure stable, reliable data acquisition, and are not universally available. A way to debottleneck the determination of key lignin parameters (including MW) is through the combination of attenuated total reflectance (ATR) infrared (IR) spectroscopy with multivariate regression. IR spectroscopy is comparatively rapid to perform, with high throughput and minimal sample preparation required, and provides an information-rich data set from which structural characteristics can be extracted through chemometrics. In essence, once a predictive model is calibrated on an appropriate data set, then it can be used to provide the desired information for future samples, saving valuable time and financial resources, based on their IR spectra alone.

**Figure 1:**
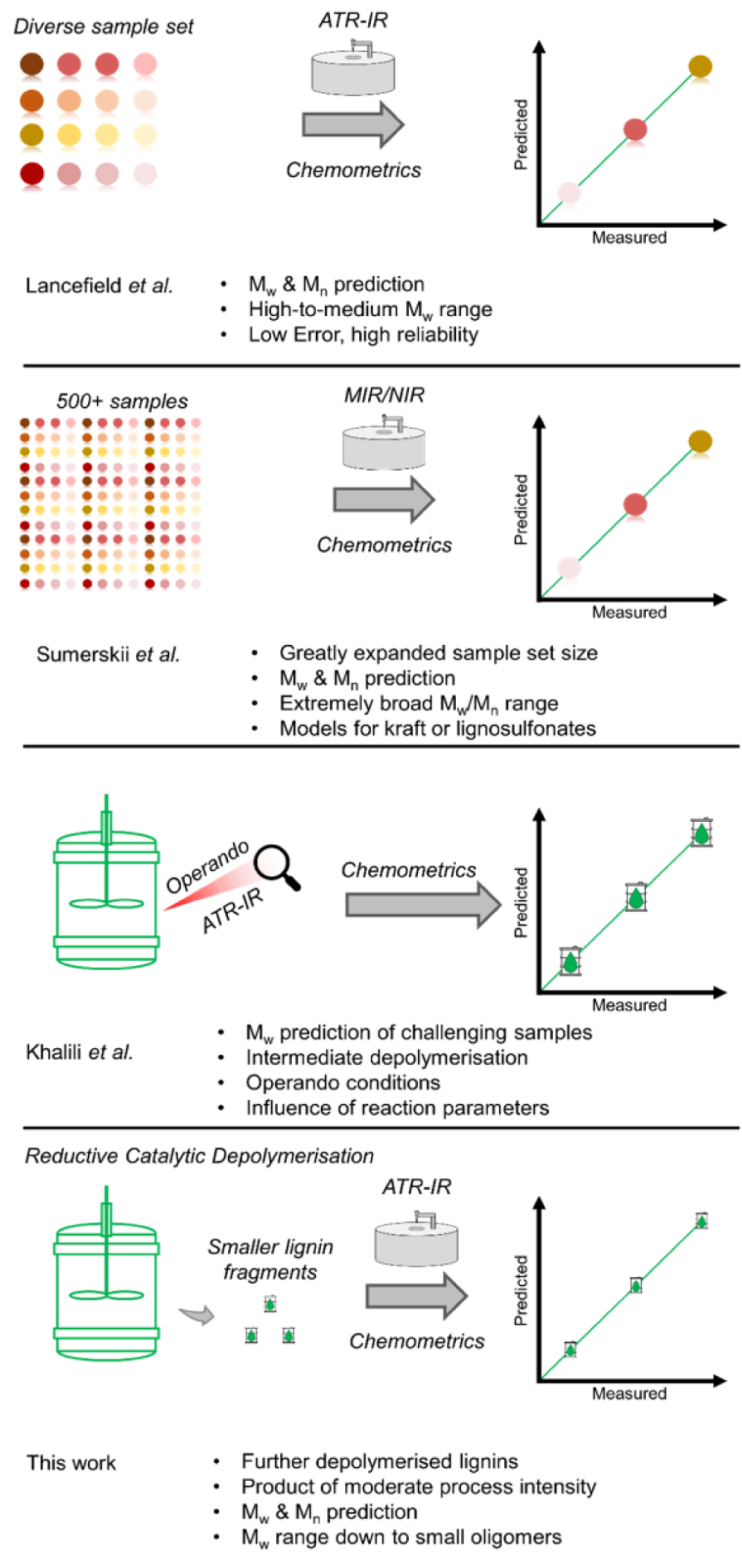
Recent advances in molecular weight prediction by ATR-IR by Lancefield et al.,^38^ Khalili et al.,^39^ and Sumerskii^40^ and in this work.

One of the key chemometric techniques is principal component analysis (PCA), which identifies features of maximal (spectral) variance in a data set known as principal components (PCs). In the context of lignin, this method has been used to construct a score plot of the samples along these PCs, allowing for the identification of trends in or clustering of samples, based purely on spectral trends or similarities, respectively.^38,39,41^ Most important perhaps is the use of partial least-squares (PLS) regression models built upon IR spectral data to predict structural (linkage abundance, [OH] content, S, G, and H units, and MW parameters)^38,41,42^ and material parameters (*T*_g_),^43^ with high speed, accuracy, and reliability. An initial study by our group, for example, used a range of weight-average molecular weight (*M*_w_) lignins as the calibration set (665–30 649 g mol^–1^) to predict the properties of a slightly narrower, although still broad MW window of samples in the validation set (1243–14 403 g mol^–1^).^38^ Very recently, Sumerskii et al. followed up on this concept with a greatly expanded sample set (500+ lignins), with models correlating either mid-IR (MIR) or near-IR (NIR) spectra with lignin structural properties (Figure 1).^40^ Functional group contents could be well-predicted in this study. The *M*_w_ range of the 500+ samples was vast, from 2170 to 38 9500 g mol^–1^ for the lignosulfonates, and from 1190 to 70 900 g mol^–1^ for the kraft lignin samples. Their models struggled, however, with MW prediction for both lignosulfonates and kraft lignin sample sets, with the exception of the model predicting *M*_n_ for the kraft samples, likely due to this incredibly broad value range and sheer diversity of the sample set.

Another key advantage of rapid spectroscopic analysis is that it also enables direct measurement of property specification of lignins and their derivatives during processing in real-time. As a result, this offers the potential of introducing process feedback loops, such that one could “tap-off” lignins with desired properties at the appropriate interval once the desired property value is reached. Recently, some of us first reported on this concept, tracking *M*_w_ and dispersity (*Đ*) during aqueous phase reforming (APR) of kraft lignin by correlating operando ATR-IR spectra with ex situ SEC data through PLS regression models.^39^ The extent of depolymerization was limited by the nature of the lignin and depolymerization process and only covered an intermediate window, down to a minimal *M*_w_ value of approximately 1800 g mol^–1^. What is not yet known is the extent to which the depolymerization can be accurately and reliably predicted, i.e., what the lower limit for the prediction of MW characteristics for lignin is. As PLS models based on IR spectra are constructed through a linear combination of wavenumber contributions relating to aromatic units, as well as interunit C–C and ether vibrations,^38,39,43^ it is anticipated that toward lower MWs, it would be more challenging to extract such information. IR-PLS methods may thus struggle with prediction of the MW of more depolymerized lignins as the relative nonuniformity of lignin dimer mixtures will lead to a lower effective concentration of the important interunit bonds, which are likely to be the pertinent descriptors for molecular weight prediction. However, it is also possible that the increased abundance of terminal functionalities may also be important for describing the lower MW lignin fragments, providing information that the PLS models may pick up on.

Here, the feasibility of determining the lower end of lignin molecular weights by IR-PLS was investigated by probing samples of depolymerized lignin (DL) of varying botanical origins subjected to varying process conditions and intensities. As a result, this sample set covers from intermediately depolymerized samples down to oligomeric lignins which center about the tetramer weight range. As *M*_n_ is (by definition) the numerical average, these lower *M*_n_ samples will thus also contain a significant quantity of yet smaller (mono-, di-, and trimeric) fragments too. Analysis by ATR-IR, followed by PCA, PLS-discriminant analysis (PLS-DA) and subsequent investigation of the PLS-DA regression vectors of the biomass classes provided valuable additional information on identifying the important wavenumbers, which contribute to the spectral variance and how these can be directly linked to their botanical classes. Rewardingly, PLS regression was able to provide accurate and reliable predictions for molecular weight characteristics also for these lower MW, (partially) depolymerized lignins for the first time. Application of this methodology to such a sample set allows for targeted MW selection, e.g. via tuning the depolymerization severity, to generate lignins better suited for a range of applications.

## Experimental Section

### Reductive Catalytic Depolymerization

All lignin and lignin oil samples were provided by VITO NV. Soluble lignin fractions were obtained by mixing lignin in methanol at 20 wt % for 4 h at 50 °C and by separating the insoluble fraction via vacuum filtration. Lignin depolymerization was carried out on the soluble lignin fractions in either a batch or continuous fashion. Batch depolymerization was performed in a 100 mL PREMEX Avalon batch reactor at 235 °C (heating ramp 10 °C min^–1^) for 2 h over 5% Pd/C (Sigma-Aldrich) with an initial H_2_ pressure of 30 bar. After 2 h of reaction, the autoclave reactor was quickly quenched with a water bath. Prior to the reactor being opened, it was flushed with N_2_ to remove the excess H_2_. The catalyst was then removed by vacuum filtration over Celite, and the filtrate was dried by rotary evaporation and in the vacuum oven at 40 °C overnight. The obtained sample was then used for further characterization.

Continuous lignin depolymerization was carried out by using a custom-built continuous flow reactor. First, the obtained soluble lignin fractions were diluted to the targeted concentrations (3, 5, and 7 wt % of lignin in methanol) through the addition of fresh methanol. The lignin solutions were pumped with flow rates of 25, 35, 50, and 100 mL h^–1^ through a fixed bed reactor. The tested reactor temperatures were 200, 215, 225, and 235 °C. The catalysts used were 1% Pd and 2% Pd on Al_2_O_3_ support (Heraeus). The back pressure regulator was set to 80 bar and the system was pressurized by means of a constant H_2_ flow of 1500 N mL h^–1^. The samples were collected over a period of 1 h, whereas the total operation time varied between 3 and 24 h. The collected samples were pre-dried under reduced pressure (rotary evaporation) and subsequently placed into the vacuum oven at 45 °C overnight. The obtained material was used for further characterization.

## Equipment

### Gel Permeation Chromatography (GPC)

GPC analysis was performed using an Agilent 1200 Series LC system equipped with a Shimadzu Prominence autosampler, a Shimadzu Prominence column oven, and a Shimadzu UV and RI detector. For the purposes of this study, only the data acquired with the UV detector was used. Depolymerized lignin samples (100 μL) were loaded on Styragel HR0.5 and HR1 columns (300 mm × 7.8 mm; 5 μm particle size), coupled in series. The elution solvent was tetrahydrofuran EMPLURA (Merck). The flow rate was 0.8 mL min^–1^, the column temperature was 40 °C, and UV detection was recorded at 254 nm. Polystyrene (PS) analytical standards (Acros Organics) with a *M*_w_ range from 162 to 19 760 g mol^–1^ were used for *M*_w_ calibration.

Size exclusion chromatography (SEC) was performed on nondepolymerised lignin samples. Here, samples were loaded on Waters Ultrahydrogel 500 (7.8 mm × 300 mm; 10 μm particle size) and Ultrahydrogel 120 (7.8 mm × 300 mm; 6 μm particle size) columns coupled in series. Size exclusion chromatography was performed at 40 °C with MQ/MeCN 80:20 + 1 M NaNO_3_, pH 11 (isocratic), at a flow rate of 0.4 mL min^–1^. Sodium polystyrenesulfonate (NaPSS) analytical standards (Agilent) with a MW range from 208 to 450 000 g mol^–1^ were used for MW calibration of polymeric lignin.

### Attenuated Total Reflectance Fourier Transform Infrared Spectroscopy (ATR-FTIR)

ATR-FTIR spectra were recorded on a PerkinElmer Spectrum Two FTIR spectrometer equipped with a PerkinElmer Universal ATR Sampling Accessory with a diamond/ZnSe plate and a LiTaO_3_ mid-IR detector. Samples of lignin and lignin oil required no particular treatment prior to measurement. Simply, a small quantity of the sample (∼5 mg) was placed onto the sample plate, ensuring the total coverage of the ATR crystal, and was pressed by the toner arm. Measurements were acquired in absorbance mode using 16 coadded scans with a resolution of 4 cm^–1^ in the range of 600–4000 cm^–1^ and with a new background spectrum recorded between each measurement. No additional corrections were made within the PerkinElmer Spectrum IR software – all preprocessing was performed within the MATLAB environment.

## Software

All basic calculations were performed in Microsoft Excel (Version 2306 Build 16.0.16529.20164). PCA and PLS regression were performed using eigenvector’s PLS_Toolbox 9.2 (24091) within the MATLAB environment R2022b (9.13.0.2126072). Graphics were built either in Microsoft Excel, in MATLAB, or in OriginPro (2021 9.8.0.200).

## Data Preprocessing and Chemometrics

The raw IR spectra were first imported into the PLS_Toolbox from the MATLAB environment. The first derivative of the data was subsequently taken, followed by a Savitsky–Golay filter (order = 2, window = 7). Then, (mean) multiplicative signal correction (MSC) was performed as a weighted normalization, followed by mean centering. When optimizing PLS/PLS-DA models, the number of LVs chosen was typically determined by a minimum in the RMSECV. If no clear minimum was observed, then the number of LVs where there was no increase in the captured variance (of the Y-block) of over 2% was chosen.

## Results and Discussion

A novel data set of DLs provided an opportunity to expand upon the MW scope of our previously reported FTIR-chemometric models.^38,39^ It is important to note that the size of a data set can have a large influence on the performance of a machine learning model, as with too few calibration samples, the machine learning model is highly susceptible to overfitting the calibration data and will poorly predict test/validation samples. On the other hand, when continuously increasing the size of the calibration set, prediction accuracy will asymptotically increase. Addition of further calibration samples may at best have no impact or at worst introduce noise into the model and impair prediction accuracy. Here, the set consisted of 62 samples, 5 of which were nondepolymerised samples and had their MW determined by SEC and the remaining 57 were DLs of varying MW values as determined by GPC. The details of the samples’ MW parameters (*M*_w_ and *M*_n_) are outlined in Table S1. The majority of the lignins were hardwood samples, with a handful of wheat straw samples as well as a few other herbaceous/grass-type lignins and two softwood lignins. Aside from the diversity in botanical origin and original characteristics (e.g. impurities and linkage content), the sample variety in the sample set resides in the differences in the depolymerization process intensity, affecting the molecular weight, as well as the fate of the original linkages.

**Figure 2:**
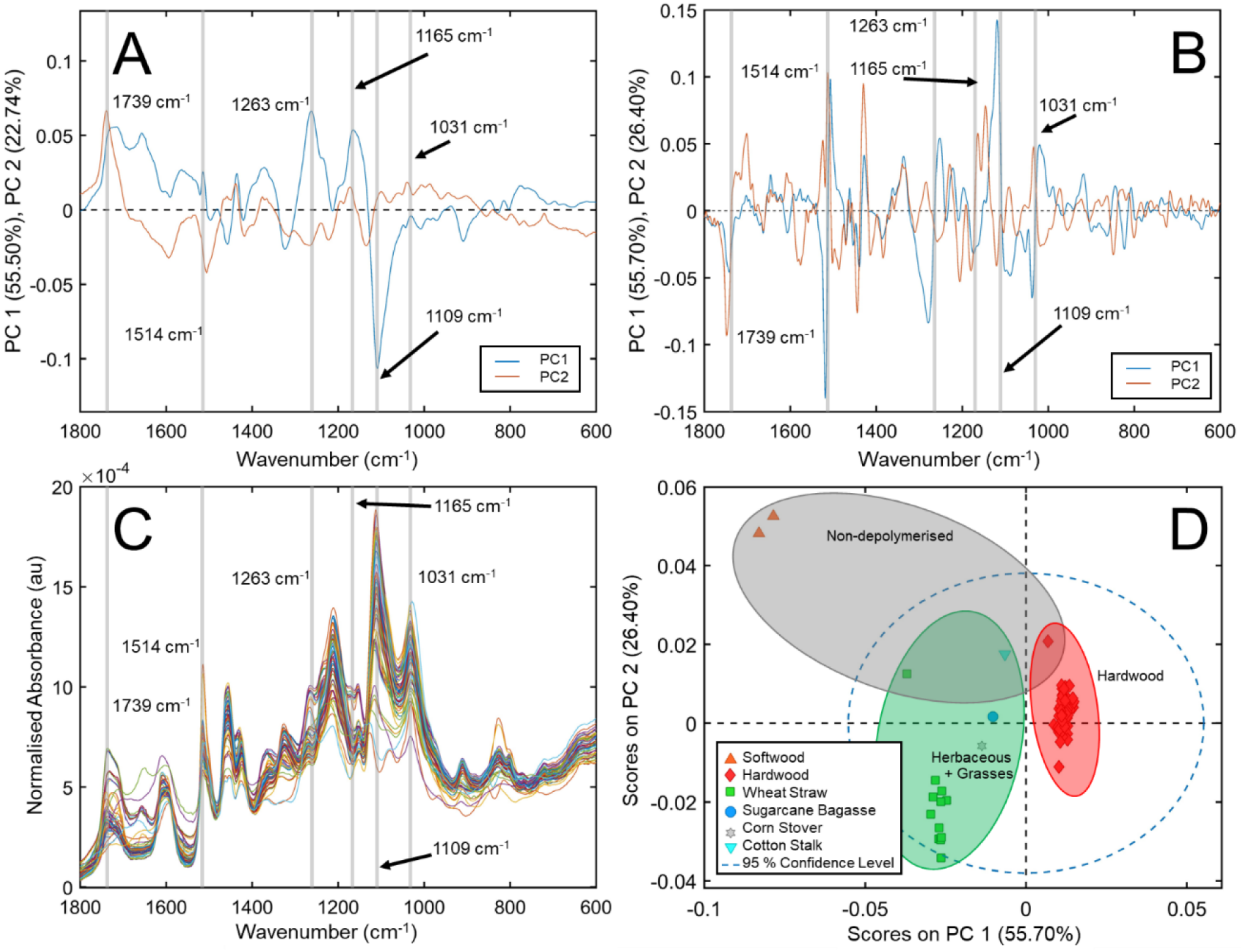
A) (Top left) PC1 and PC2 loading vectors of the set without application of the 1st derivative or SG filter. B) (Top right) PC1 and PC2 loading vectors with the full set of preprocessing settings applied. C) (Bottom left) Truncated view of (1-norm) normalized ATR-IR spectra. D) Score plot of PC1 vs PC2. Key bands have been highlighted with a gray bar in (A–C). In D), ovals have been added to visually denote the broader sample sets. For all figures, the full sample set of 62 lignins was used, and their full spectral range (4000–600 cm^–1^) of the ATR-IR was used. For (B–D), the spectra were preprocessed by taking the 1st derivative, followed by application of an SG filter and MSC, and then mean centering the data. For panel (A), the same preprocessing was used except that the 1st derivative spectra were not used and the SG filter was not applied for ease of visualizing important wavenumbers.

**Figure 3:**
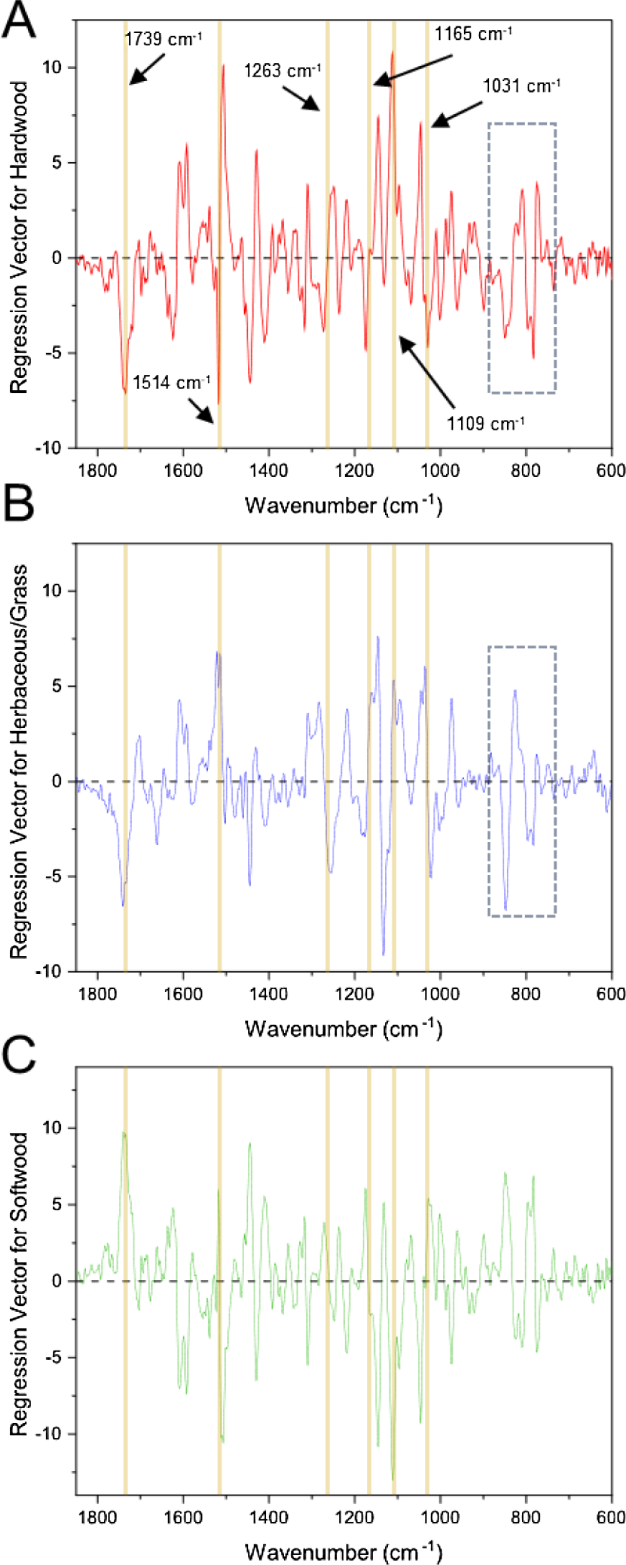
Zoomed in view of PLS-DA model regression vectors in the range of 1850–600 cm^–1^ for A) hardwood, B) herbaceous/grass, and C) softwood. Five LVs were used, and the standard preprocessing settings were applied (1st derivative, with SG filter, MSC, and then mean centering). Highlighted bands are those identified from PCA (Figure 2). Gray boxes indicate an example region of differing features between (A) and (B). Full regression vectors, the PLS-DA score plot, and the details of the PLS-DA model are available in Figures S1 and S2 and Table S3, respectively.

The DL (oil) samples were used “as-is” for ATR-IR spectral acquisition, i.e., no additional sample preparation was required, and no particular storage method was used. The obtained samples were all preprocessed prior to any statistical analysis. First, a first order derivative with a Savitzky–Golay (SG) filter was applied, effectively performing as a baseline correction in addition. Following this, multiplicative signal correction (MSC) was applied to normalize the data, and finally, the spectra were mean-centered. Investigation of the loading vectors of PC1 and PC2 was performed with the aforementioned preprocessing steps (Figure 2b), and also without application of first order derivative correction, as we reported previously, to assist with assignment of peaks (Figure 2a).^43^ Comparison of these plots with the normalized IR spectra (Figure 2c) allowed for the identification of the peaks and thus functionalities contributing most to the variation within the data set (Table 1). The complexity of the lignin structure, and thus the spectrum, leads to signals with compounded contributions, which cannot always be easily deconvoluted. It is thus important to reiterate that these vibrational assignments must remain tentative, and they only highlight the most important contributors to the peaks in IR spectra of this data set of lignins. This does not, however, prevent one from relating key spectral features to these more general structural motifs. In this case, the peaks of interest were those which manifested as strongly positively or negatively loaded in the PC loading vectors, as they are thus important for describing spectral variance. It is also important to note that when interpreting PCs resulting from modeling derivative spectra, much like the derivative spectra themselves, the peaks are split into a positive and negative peak couple. Hence, the gray bars denoting assignable peaks (based on the comparison of Figure 2a and Figure 2c) fall (approximately) at the center point of the peak couples within Figure 2b. For instance, the relative intensities of the peaks at 1263 and 1109 cm^–1^, which have been shown to correlate with the S/G ratio,^44^ are present with positive and negative weights, respectively, within the PC1 loading vector. Indeed, this is well corroborated by the score plot shown in Figure 2d. Increasing S-ring content and (relatively) decreasing G-ring content were found to correlate with increasingly positive scores on PC1, with softwood lignins showing the most negative score, herbaceous/grasses being slightly negative, and hardwoods being positive. In essence, the samples are clustered by botanical origin, as was previously also found for technical lignin sample sets of varying botanical origin.^38,41−43^ The score plot also reveals that there is a somewhat distinct separation of the nondepolymerised samples from the others, with all five showing the most positive scores on PC2 (relative to their cluster) and being somewhat distinct from the hardwood and wheat straw clusters. Additionally, the corn stover and sugar cane bagasse samples were spatially separated from the wheat straw cluster, showing a less negative score on PC1, further showing the sensitivity of PCA to the lignin’s original feedstock. A method to provide direct insight into which spectral features have the highest weighting for correct classification is PLS-discriminant analysis (PLS-DA), which has been successfully applied to spectral data sets of (for example) plastics and whole biomass samples previously.^45,46^ Here, this method was performed by correlating each sample’s spectra with their associated broader biomass types (softwood, hardwood, or herbaceous/grass). Interrogation of the resulting regression vectors therefore directly provided information about the weighting of each of the wavenumbers (Figure 3). The regression vectors of hardwood and herbaceous/grass classes showed similar (visual) line shape, but subtle differences do emerge upon closer inspection, for example, in the 750–900 cm^–1^ region, as highlighted by the gray boxes in Figure 3a,b. By contrast, the softwood regression vector was found to be almost completely opposite to these two more similar regression vectors with clear differences across the range of wavenumbers. Many of the major discrepancies (relative to the hardwood and herbaceous/grass classes, respectively) manifest as an inversion of the peak couples surrounding the highlighted wavenumbers from PCA (Figure 2a–c), as shown by the yellow bars in Figure 3a–c. PLS-DA thus shows that the spectral variance contribution for this sample set is still dominated by botanical origin differences and that the influence of the process intensity on the spectra is, by comparison, relatively minor. Further information relating to the PLS-DA models can be found in Table S3.

**Table 1:** Key Assignments of ATR-IR Spectra in the Range of 1800-600 cm^–1^ by Cross-Comparison with the Associated Bands Seen in PC1 and PC2 Eigenspectra without and with 1st Derivative Preprocessing

wavenumber (cm^–1^)^a^	assignment
1737	C=O stretch
1514	aromatic skeletal vibration
1263	G-ring related ring breathing + C–O stretch
1165	H, G, and S related band
1109	C–O related stretch common in both S and G rings
1031	C–O related stretch common in G-type lignins

a Wavenumbers correspond to peaks from the IR spectra with a corresponding peak couple in their PCs.

Individual PLS regression models were calibrated to predict MW parameters (*M*_n_ and *M*_w_) as quantified by GPC, by their correlation with the samples’ FTIR spectra. Data preprocessing parameters were optimized with the aim of minimizing the root mean squared error of cross-validation (RMSECV) to generate the most reliable model. Additionally, minimization of the RMSECV/RMSEC ratio (where RMSEC is the root mean squared error of calibration) ensures that the model is less susceptible to overfitting. Initial model parameters were selected on the basis of previous work from within our group on different lignin samples, but were optimized for the sample set used herein (Table S2).^38,39,43^ Furthermore, the choice of latent variables (LVs) was a key step in the creation of the predictive models as too few may not adequately describe the calibration data set, but too many may lead to overfitting. Here, the optimal number of LVs was determined, similar to the data preprocessing, by minimization of RMSECV and further by ensuring the RMSECV/RMSEC ratio was close to unity. A particular optimization step that differed for the targeted MW property was the use of slightly different spectral ranges to yield optimal models.

**Figure 4:**
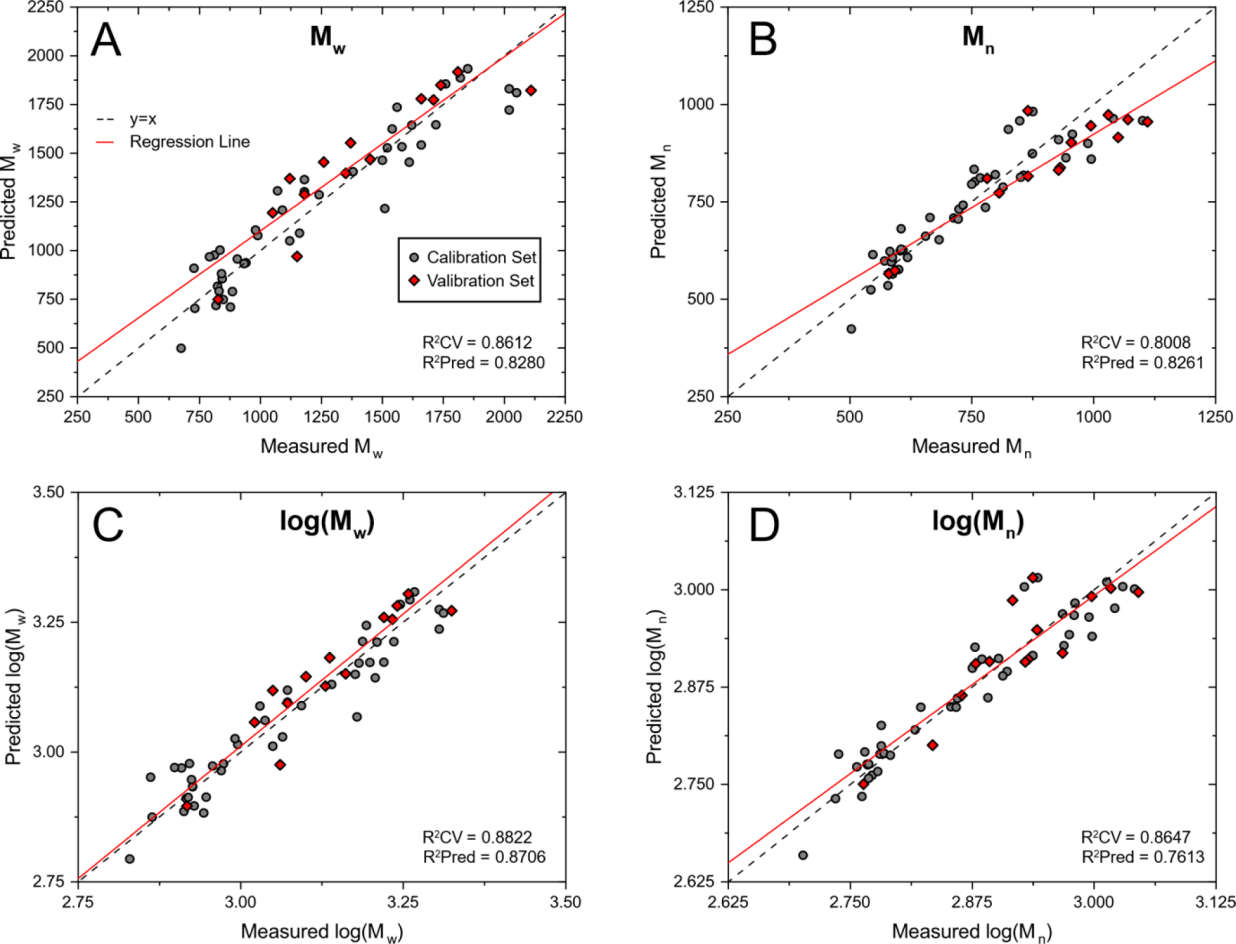
Scatterplots showing the results of PLS regression between MW characteristics: A) *M*_w_, B) *M*_n_, C) log(*M*_w_), and D) log(*M*_n_) as measured by GPC and the predicted values as obtained from the ATR-IR spectra of the lignin oil samples. 57 samples were utilized in total and were split into a Cal set (43 samples, gray circles) and a Val set (14 samples, red diamonds). The regression line shown was plotted only through the predicted data points.

The MW characteristics of the DLs were measured by GPC using tetrahydrofuran as the eluent, except for the nondepolymerized samples. They were unfortunately not soluble in the GPC eluent, so their MWs were instead quantified on an alkaline GPC system (Table S1). Given the general challenges of interinstrument MW comparison (particularly when changing the method and eluent), these samples were therefore excluded from the PLS modeling in each case. When optimizing the preprocessing for PLS models to predict these MW characteristics, the models obtained high *R*^2^ CV, low RMSECV, and low RMSECV/RMSEC ratios (Table 2). 57 samples were thus used in total, and to validate the models, the sample set was split into calibration (Cal) and validation (Val) sets to achieve a Cal:Val ratio of 75:25 resulting in 43 Cal samples and 14 Val samples. A Kennard–Stone (KS) algorithm operating on a Mahalanobis distance was utilized to ensure a uniform split of the samples on the basis of their (preprocessed) FTIR spectra.^47^ A full overview of the model settings and the results of optimization of each parameter are provided in Table S4. Also, as each of the parameters (*M*_w_, *M*_n_, Log(*M*_w_), and Log(*M*_n_)) required different preprocessing conditions, separate splits were performed for each parameter, as has been outlined in Table S5. Despite the challenge of working with depolymerized lignin samples, PLS regression yielded surprisingly good prediction of molecular weight characteristics (Table 2). Modeling of *M*_w_ was found to provide more favorable RMSECV, *R*^2^ CV, RMSEP, and relative error (RE) values than when predicting *M*_n_ (Figure 4 and Table 3). This, as well as the relatively large bias in the regression line for the *M*_n_ model, is in contrast to the observations by Sumerskii et al. and as has been reported previously in our group for kraft lignin-based models.^38,40^ The use of the logarithm of *M*_w_ and *M*_n_ was found to provide higher *R*^2^ CV values and similar RMSECV/RMSEC (Table 3), indicating that there is an improvement in the model’s reliability and no significant change in the degree of overfitting. The reduced RE in each case is due to linearization of the molecular weight values and thus the error, as has been reported by our group previously.^38,43^ To our surprise, however, when changing from *M*_n_ to log(*M*_n_), a decrease in *R*^2^ Pred was found and observed, in spite of the similarly large increase in *R*^2^ CV. Importantly, all models showed low RMSECV/RMSEC ratios, suggesting that an appropriate number of LVs were selected for the modeling in each case. The REs of all models (predicting *M*_n_, *M*_w_, log(*M*_n_), and log(*M*_w_)) were found to be higher than those of the corresponding models reported for nondepolymerized kraft-only sample sets,^38^ although here, the RE values are still well within acceptable limits of between 9.9% and 11.2%.

**Table 2:** Optimal Preprocessing Settings for Molecular Weight Characteristics Determined by GPC

			variance (%)				calibration		cross-validation				
entry	feature	value range	*X*	*Y*	spectral width (cm^–1^)	LVs	RMSEC	*R*^2^ Cal	RMSECV	CV Bias	*R*^2^ CV	RMSECV/RMSEC	RE (%)
1	*M*_w_^a^	1435	91.17	84.36	4000–2650; 1850–750	4	62.01	0.86	68.88	1.81	0.83	1.11	11.35
2	*M*_n_^a^	607	91.92	88.83	2000–750	3	134.26	0.89	150.78	1.56	0.86	1.12	10.51
3	Log(*M*_w_)	0.495	93.49	92.94	2000–750	4	0.03683	0.93	0.04866	–5.43 × 10^–4^	0.88	1.32	9.83
4	Log(*M*_n_)	0.344	93.51	89.12	4000–600	4	0.03117	0.89	0.03521	8.84 × 10^–4^	0.86	1.13	10.24

a Units: g mol–^1^. For *M*_n_, *M*_w_, log(*M*_n_), and log(*M*_w_), 1st derivative spectra were taken, and an SG filter was applied, followed by MSC and mean centering. RE = RMSECV/range.

**Table 3:** Results of PLS Regression between MW Characteristics as Determined by GPC and ATR-IR Spectra of the DL Samples^a^^,^^b^

		variance (%)		calibration		cross-validation		prediction			
feature	range	*X*	*Y*	RMSEC	R^2^ Cal	RMSECV	*R*^2^ CV	RMSEP	*R*^2^ Pred	RE (%)	RMSECV/RMSEC
*M*_w_	1435	91.24	89.69	131.67	0.8969	152.96	0.8612	153.89	0.828	10.66	1.16
*M*_n_	607	93.07	85.75	57.34	0.8575	67.9	0.8008	84.03	0.8261	11.19	1.18
log(*M*_w_)	0.82	91.11	91.39	0.042	0.9139	0.0492	0.8822	0.044	0.8706	9.93	1.17
log(*M*_n_)	0.49	93.21	90.6	0.0297	0.906	0.0357	0.8647	0.0372	0.7613	10.4	1.2

a Models predicting *M*_w_ and log(*M*_w_) utilized 3 LVs. *M*_n_ and log(*M*_n_) were predicted using 4 LVs. RE = RMSECV/range.

b 57 samples were utilized in total and were split into a Cal set (43 samples) and a Val set (14 samples).

**Figure 5:**
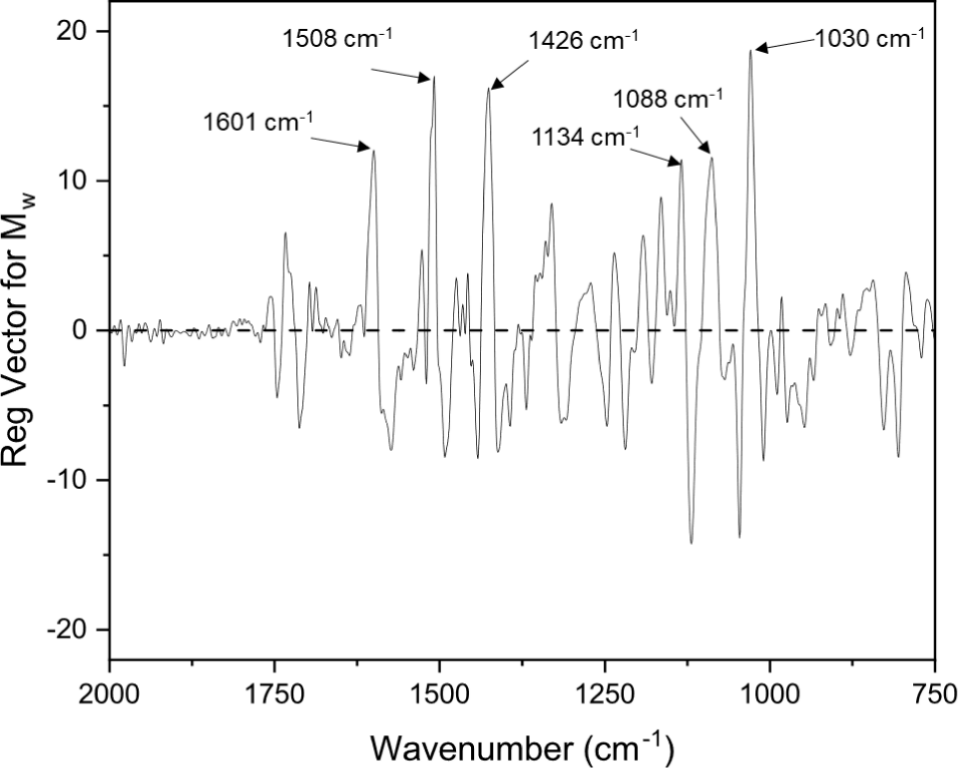
Regression vector for the general PLS model correlating the 57 lignin IR spectra with their *M*_w_ using 3 LVs.

It was also observed that the regression vectors of the PLS models are all (visually) similar. Figure 5 shows a representative (PLS) regression vector, correlating the IR of the 57 DL samples with *M*_w_ (Table S2, entry 5); for other regression vectors (*M*_n_, log(*M*_n_), and log(*M*_w_)), see Figure S3. Some of the key features relate to the wavenumbers where there is greater structural variance, as extracted from PCA (performed for the entire sample set; Figure 2 and Table 1). Specifically, the features at 1030 cm^–1^ (G ring related), 1134 cm^–1^ (S and G ring related), and 1508 cm^–1^ (aromatic skeletal vibration) are common between the first two PCs from PCA, and the aforementioned regression vector of the *M*_w_-predicting PLS model. However, some of the more hidden features of the spectra, which do not vary greatly within the sample set but are also key predictors in determining larger *M*_w_ values, are those at 1601 cm^–1^ (aromatic skeletal vibration), 1426 cm^–1^ (combined aromatic skeletal vibration and C–H in-plane deformation), and 1088 cm^–1^ (C–O vibration in 2° alcohols and aliphatic ethers). Most of these bands are indeed related to aromatic content within the lignin; however, the band at 1088 cm^–1^ is related to the presence of aliphatic alcohols (and any remaining ether linkages) instead. Generally, within IR spectra, this C–O stretching frequency has strong absorbance, but with these hardwood lignin samples, it is more challenging to identify due to the strong aromatic C-to-oxygen band at 1109 cm^–1^ (Figure 2c) occluding this feature.

To try to further push the performance of the PLS models and reduce the RE, the sample set was restricted to a more refined set of samples of only hardwood samples (44 lignins) to help eliminate some of the botanical-origin-derived spectral variance, which otherwise likely acts as noise for MW-predicting models. As above, the sample set was split by a KS algorithm operating on Mahalanobis distance to achieve a Cal:Val split of 75:25 such that Cal = 33 samples and Val = 11 samples. As anticipated, all models showed a decrease in RMSEP compared to the original data set, although the improvement was relatively subtle. Additionally, there was an accompanying reduction in RMSEC for all models, and an increase in RMSECV for *M*_n_ and log(*M*_n_) models and decrease for *M*_w_ and log(*M*_w_) models (Table S4). In total, all models have a slightly increased RE, which, combined with the increased RMSECV/RMSEC ratio, is indicative of a slight increase in overfitting. This was a surprising observation, given that in previous works, smaller, more focused Cal sets have managed to predict MW characteristics with lower RE and RMSECV/RMSEC, respectively.^38,43^ Here, however, a larger sample set is perhaps required to achieve similar accuracy and reliability.

## Conclusion

The results presented herein extend the scope of the ATR-IR/chemometrics-based rapid characterization method for lignin MW characterization to less “lignin-like” DLs. Thus, it provides an extension to the growing chemometric toolbox that is essential for the low-time and low-cost analysis of lignin and complex lignin-derived product matrices. It was found here that similar to typical technical lignins, the DLs were well (spatially) separated using PCA on their ATR-IR spectra, displaying clustering of the samples by botanical origin. The key spectral features that are most salient for classification were highlighted by PLS-DA, and it was shown how these connect with the structural features.

Rewardingly, the IR-PLS methodology proved to still provide accurate predictions for MW parameters for such small chain DLs. These accurate models showed RE values between 9.9% and 11.2%, which is only a moderate increase in error when compared to a previous example of MW prediction using IR-PLS on nondepolymerised lignins.^38^ Intriguingly, simplification of the data set to only hardwood samples did not improve the model reliability or accuracy any further, resulting in slightly more accurate but less reliable models. Interrogation of the PLS regression vectors also unveiled the key spectral descriptors for the prediction of the MW properties. Consequently, this has implications that in further studies, models may still be able to predict MW down to the monomer–dimer range, although the exact limit is not yet known.

As these lignin processing steps are increasing in popularity, DLs are seeing more widespread use as feedstock, and a significant body of research is now dedicated toward the production of a wide range of lignin-derived products using them. As such, appropriate and convenient analytical methods must be developed alongside to cope with the complexity of these samples. To the best of our knowledge, the herein described extension of the IR-chemometric approach is the first implementation for qualitative and quantitative characterization of heavily processed (depolymerized) lignins and lignin oils. Additionally, it is a further stepping stone toward more widespread implementation of this powerful approach into different stages of the lignin-processing value chain. The next step beyond this work is to construct PLS models that predict the hydroxyl group content/distribution for such sample sets, as well as test for the feasibility of shifting from isolated lignin samples to samples that are in solution, more closely mimicking the depolymerization process conditions.

## Supporting Information Available

A supporting document containing additional figures, as well as tables containing sample and model characteristics is provided. Overview of (depolymerized) lignin sample information and molecular weight features (Table S1); optimization parameters for PLS models for the set of 57 lignin samples (Table S2); regression vectors for the PLS-DA model (Figure S1); PLS-DA score plot (Figure S2); results of PLS regression between MW characteristics (Table S4); validation samples for PLS regression models with hardwood-only samples (Table S5); full regression vectors for PLS models (Figure S3). (PDF)

## Abbreviations

IR: infrared
ATR: attenuated total reflectance
PCA: principal component analysis
PLS: partial least-squares
PLS-DA: partial least-squares discriminant analysis
MW: molecular weight
Mw: weight-average molecular weight
Mn: number-average molecular weight
