# Supplementary material for: Predicting Molecular Weight Characteristics of Reductively Depolymerized Lignins by ATR-FTIR and Chemometrics
Source: ACS Sustain Chem Eng. 2024 May 29;12(23):8968–77. doi: 10.1021/acssuschemeng.4c03100 (PMC11167637; doi:10.1021/acssuschemeng.4c03100)
Supplement: Supplementary file 1 — sc4c03100_si_001.pdf [file sc4c03100_si_001.pdf]

Supporting Information:

Predicting Molecular Weight Characteristics of Reductively Depolymerised Lignins by ATR-FTIR and Chemometrics

Luke A. Riddell[a], Peter de Peinder[b],[c], Viviana Polizzi[d], Karolien Vanbroekhoven[d], Florian Meirer[b],*, Pieter C. A. Bruijnincx[a],*

[a] L.A. Riddell, P.C.A. Bruijnincx
Utrecht University, Organic Chemistry & Catalysis, Institute for Sustainable and Circular Chemistry, Faculty of Science, 3584 CG Utrecht (The Netherlands); Email: p.c.a.bruijnincx@uu.nl

[b] F. Meirer, P. de Peinder
Utrecht University, Inorganic Chemistry & Catalysis, Institute for Sustainable and Circular Chemistry, Faculty of Science, 3584 CG Utrecht (The Netherlands); Email: f.meirer@uu.nl

[c] P. de Peinder
VibSpec, Haaftenlaan 28, 4006 XL Tiel (The Netherlands)

[d] V. Polizzi, K. Vanbroekhoven
Sustainable Polymer Technologies team, Materials & Chemistry unit, Flemish Institute for Technological Research (VITO), 2400 Mol (Belgium)

Number of Pages: 8

Number of Tables: 5

Number of Figures: 3

Contents

Supporting Information: 1
Lignins 3
Pre-Processing Optimisation 5
Additional PLS-DA/PLS Regression Information 6

Lignins

TableS1. Overview of (depolymerised) lignin sample information and molecular weight features.

Lignin Sample Code	Biomass Type	Class	Depolymerisation Method	Mw[a]	Mn[a]	Log(Mw)	Log(Mn)
1	hardwood	hardwood	batch	1120	713	3.0	2.9
2	sugarcane bagasse	herbaceous/ grass	batch	1160	722	3.1	2.9
3	corn stover	herbaceous/ grass	batch	941	655	3.0	2.8
4	hardwood	hardwood	batch	1510	814	3.2	2.9
5	wheat straw	herbaceous/ grass	continuous	811	571	2.9	2.8
6	wheat straw	herbaceous/ grass	continuous	843	586	2.9	2.8
7	wheat straw	herbaceous/ grass	continuous	824	578	2.9	2.8
8	wheat straw	herbaceous/ grass	continuous	827	580	2.9	2.8
9	wheat straw	herbaceous/ grass	continuous	830	585	2.9	2.8
10	wheat straw	herbaceous/ grass	continuous	834	587	2.9	2.8
11	wheat straw	herbaceous/ grass	continuous	848	592	2.9	2.8
12	wheat straw	herbaceous/ grass	continuous	877	600	2.9	2.8
13	wheat straw	herbaceous/ grass	batch	905	603	3.0	2.8
14	wheat straw	herbaceous/ grass	batch	1150	683	3.1	2.8
15	hardwood	hardwood	batch	1180	756	3.1	2.9
16	hardwood	hardwood	batch	1180	767	3.1	2.9
17	hardwood	hardwood	batch	1180	755	3.1	2.9
18	wheat straw	herbaceous/ grass	batch	933	609	3.0	2.8
19	hardwood	hardwood	continuous	675	503	2.8	2.7
20	hardwood	hardwood	continuous	731	543	2.9	2.7
21	hardwood	hardwood	continuous	818	587	2.9	2.8
22	hardwood	hardwood	continuous	885	618	2.9	2.8
23	hardwood	hardwood	continuous	1610	750	3.2	2.9
24	hardwood	hardwood	continuous	1660	798	3.2	2.9
25	hardwood	hardwood	continuous	1710	825	3.2	2.9
26	hardwood	hardwood	continuous	1760	848	3.2	2.9
27	hardwood	hardwood	continuous	1850	875	3.3	2.9
28	hardwood	hardwood	continuous	1810	865	3.3	2.9
29	hardwood	hardwood	continuous	727	547	2.9	2.7
30	hardwood	hardwood	continuous	791	582	2.9	2.8
31	hardwood	hardwood	continuous	840	605	2.9	2.8
32	hardwood	hardwood	continuous	1090	724	3.0	2.9
33	hardwood	hardwood	continuous	1240	806	3.1	2.9
34	hardwood	hardwood	continuous	1380	865	3.1	2.9
35	hardwood	hardwood	continuous	1500	931	3.2	3.0
36	hardwood	hardwood	continuous	2020	1050	3.3	3.0

37	hardwood	hardwood	continuous	1070	778	3.0	2.9
38	hardwood	hardwood	continuous	1260	856	3.1	2.9
39	hardwood	hardwood	continuous	1450	928	3.2	3.0
40	hardwood	hardwood	continuous	1560	956	3.2	3.0
41	hardwood	hardwood	continuous	1660	994	3.2	3.0
42	hardwood	hardwood	continuous	1740	1040	3.2	3.0
43	hardwood	hardwood	continuous	1820	1030	3.3	3.0
44	hardwood	hardwood	continuous	2020	1070	3.3	3.0
45	hardwood	hardwood	continuous	2110	1110	3.3	3.0
46	hardwood	hardwood	continuous	1580	995	3.2	3.0
47	hardwood	hardwood	continuous	1350	850	3.1	2.9
48	hardwood	hardwood	continuous	1540	928	3.2	3.0
49	hardwood	hardwood	continuous	1620	954	3.2	3.0
50	hardwood	hardwood	continuous	1720	988	3.2	3.0
51	hardwood	hardwood	continuous	2050	1100	3.3	3.0
52	hardwood	hardwood	continuous	989	605	3.0	2.8
53	hardwood	hardwood	continuous	980	664	3.0	2.8
54	hardwood	hardwood	continuous	1050	732	3.0	2.9
55	hardwood	hardwood	continuous	1120	781	3.0	2.9
56	hardwood	hardwood	continuous	1370	874	3.1	2.9
57	hardwood	hardwood	continuous	1520	943	3.2	3.0
58[b]	softwood	softwood	non-modified	2720	917	3.4	3.0
59[b]	hardwood	hardwood	non-modified	4330	1070	3.6	3.0
60[b]	cotton stalks	herbaceous/ grass	non-modified	9290	1890	4.0	3.3
61[b]	wheat straw	herbaceous/ grass	non-modified	7610	1420	3.9	3.2
62[b]	softwood	softwood	non-modified	1350	697	3.1	2.8

[a]: Units g mol^{-1}, [b]: Non-depolymerised lignins, which, for molecular weight determination, were measured by alkaline GPC as detailed in the Methods & Materials section.

Pre-Processing Optimisation

Table S2. Optimisation parameters for PLS models for the set of 57 lignin samples.

Entry	Parameter	Range	Variance X	Variance Y	Spectral Window (cm^{-1})	LVs	RMSEC	RMSECV	Bias	CV Bias	R^2 Cal	R^2 CV	RE[a] (%)
1	Mn	607	95.62	93.11	4000-600	4	44.10	64.65	0	3.19	0.93	0.86	10.65
2	Mn	607	91.69	84.98	2000-750	3	65.14	70.76	0	2.64	0.85	0.82	11.66
3[b]	Mn	607	91.17	84.36	4000-2650; 1850-750	4	62.01	68.88	0	1.81	0.86	0.83	11.35
4	Mw	1435	90.96	88.45	4000-600	3	136.56	152.70	0	2.51	0.88	0.86	10.64
5[b]	Mw	1435	91.92	88.83	2000-750	3	134.26	150.78	-2.27E-13	1.56	0.89	0.86	10.51
6	Mw	1435	91.27	88.39	4000-2650; 1850-750	3	136.91	152.85	0	2.42	0.88	0.86	10.65
7	Log(Mw)	0.49	90.87	90.61	4000-600	4	0.04247	0.04968	0	0.00	0.91	0.87	10.04
8b]	Log(Mw)	0.49	93.49	92.94	2000-750	4	0.03683	0.04866	0	0.00	0.93	0.88	9.83
9	Log(Mw)	0.49	91.18	90.57	4000-2650; 1850-750	4	0.04254	0.04967	0	0.00	0.91	0.87	10.03
10[b]	Log(Mn)	0.34	93.51	89.12	4000-600	4	0.03117	0.03521	0	0.00	0.89	0.86	10.24
11	Log(Mn)	0.34	93.56	89.59	2000-750	4	0.03050	0.03596	-4.44E-16	0.00	0.90	0.86	10.46
12	Log(Mn)	0.34	93.87	88.94	4000-2650; 1850-750	4	0.03143	0.03534	0	0.00	0.89	0.86	10.28

[a] RE = RMSECV/parameter range [b] Entries represent the optimal model conditions for use in predictive modelling in the main body of work.

Additional PLS-DA/PLS Regression Information

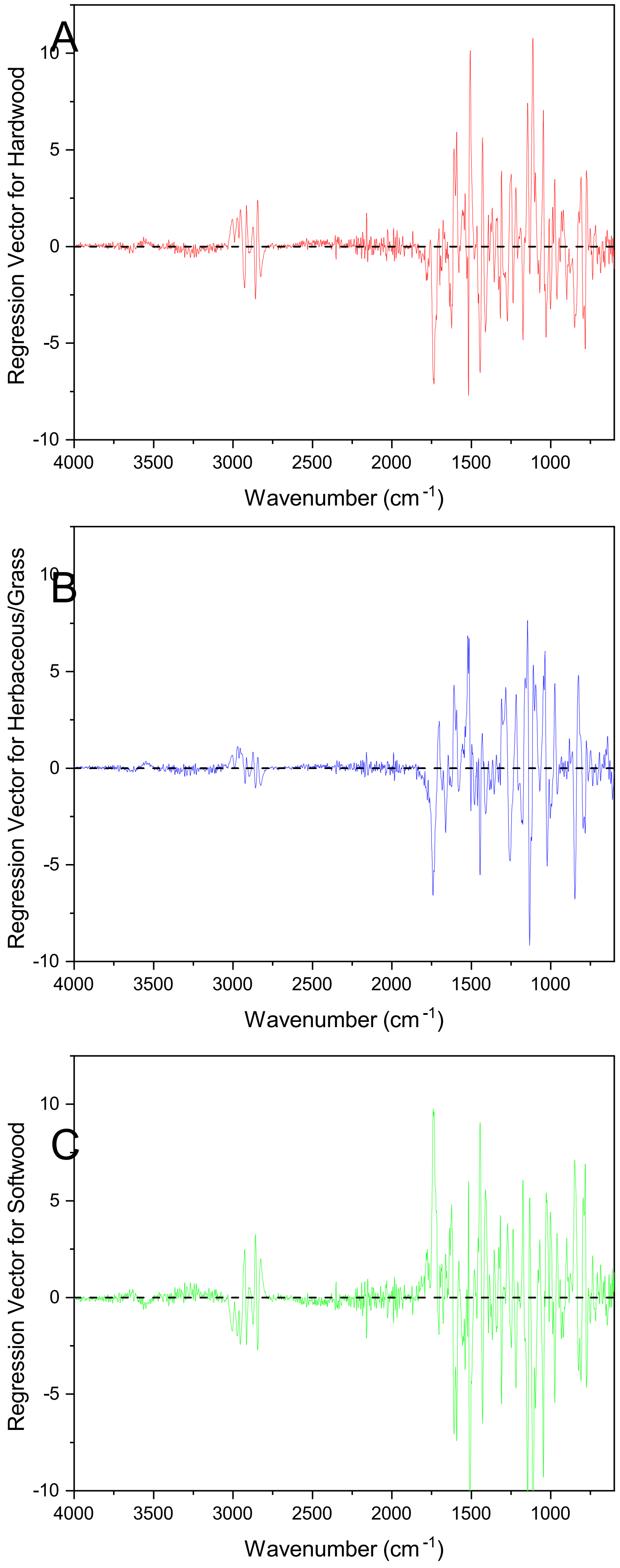

Figure S1. Regression vectors for PLS-DA model built on the sample set of 62 lignins for classes a) Hardwood, b) Herbaceous/grass, and c) Softwood. The model was constructed with 5 LVs and used the same pre-processing as detailed in the Methods and Materials section.

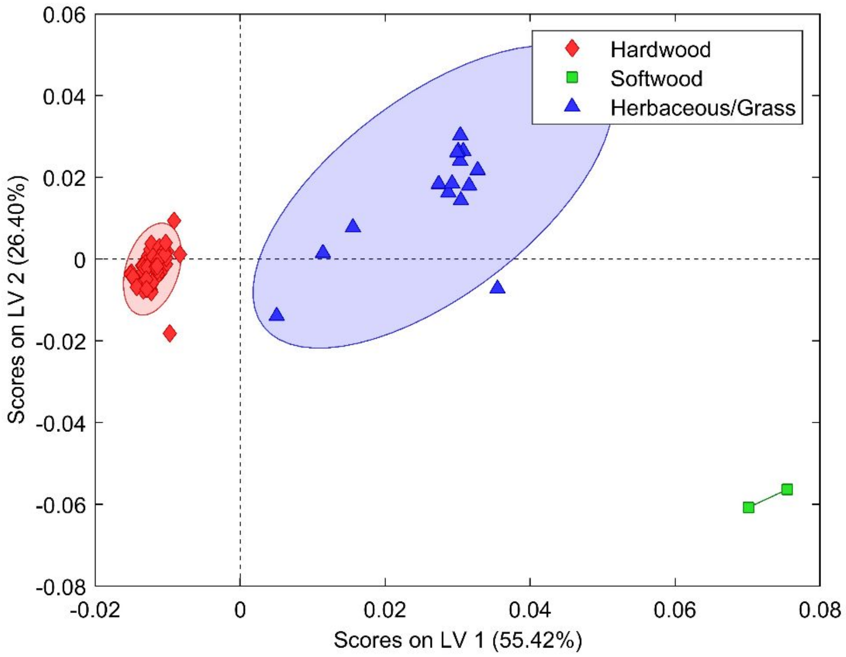

Figure S2. PLS-DA score plot of the total set of 62 lignin samples. The model was constructed with 5 LVs and used the same pre-processing as detailed in the Methods and Materials section.

Table S3. PLS-DA models constructed by regression between the FTIR spectra of the 62 DL samples and their corresponding broad biomass classes.

	Biomass Class		
**Metric**	**Hardwood**	**Softwood**	**Grass**
Sensitivity (Cal)	1	1	1
Specificity (Cal)	1	1	1
Sensitivity (CV)	1	1	0.933
Specificity (CV)	0.941	1	1
Class Error (Cal)	0	0	0
Class Error (CV)	0.0294	0	0.0333
RMSEC	0.0408	0.0105	0.0406
RMSECV	0.1184	0.0330	0.1376
Bias	0	6.94E-18	-2.78E-17
CV Bias	-0.0008	-0.0015	0.0023
R^2Cal	0.9917	0.9965	0.9910
R^2CV	0.9321	0.9836	0.8980

5 LVs were used and a total of 92.87 % of X-block (FTIR) variance was captured, explaining 99.30 % of Y-block information (Biomass classes)

Table S4. Results of PLS regression between MW characteristics as determined by GPC and ATR-IR spectra of the hardwood lignin oil samples. 44 samples were utilised in total and were split into a Cal set (33 samples) and a Val set (11 samples).

		Variance (%)		Calibration		Cross-Validation		Prediction			
**Feature**	**Range**	**X**	**Y**	**RMSEC**	**R^2 Cal**	**RMSECV**	**R^2 CV**	**RMSEP**	**R^2 Pred**	**RE[b] (%)**	**RMSECV/ RMSEC**
M_w	1229	69.72	96.90	66.90	0.9690	149.55	0.84949	126.902	0.8572	12.17	2.24
M_n	597	88.5	94.06	38.85	0.9406	72.69	0.80267	59.4681	0.7892	12.18	1.87
$\log(M_w)$	0.49498	77.33	96.53	0.02453	0.9653	0.05076	0.85584	0.087239	0.87239	10.26	2.07
$\log(M_n)$	0.33982	85	93.88	0.02233	0.9388	0.0389	0.8221	0.031458	0.7983	11.46	1.74

Models predicting M_w and $\log(M_w)$ utilised 3 LVs. M_n and $\log(M_n)$ were predicted using 4 LVs. RE = RMSECV/range.

Table S5. Validation samples for PLS regression models with hardwood-only samples, where 44 samples were utilised in total and were split into a Cal set (33 samples) and a Val set (11 samples).

Validation Samples	
M_w & log(M_w)	Mn & log(Mn)
27	27
28	35
30	39
35	41
36	44
39	45
40	47
42	49
44	53
47	54
49	57

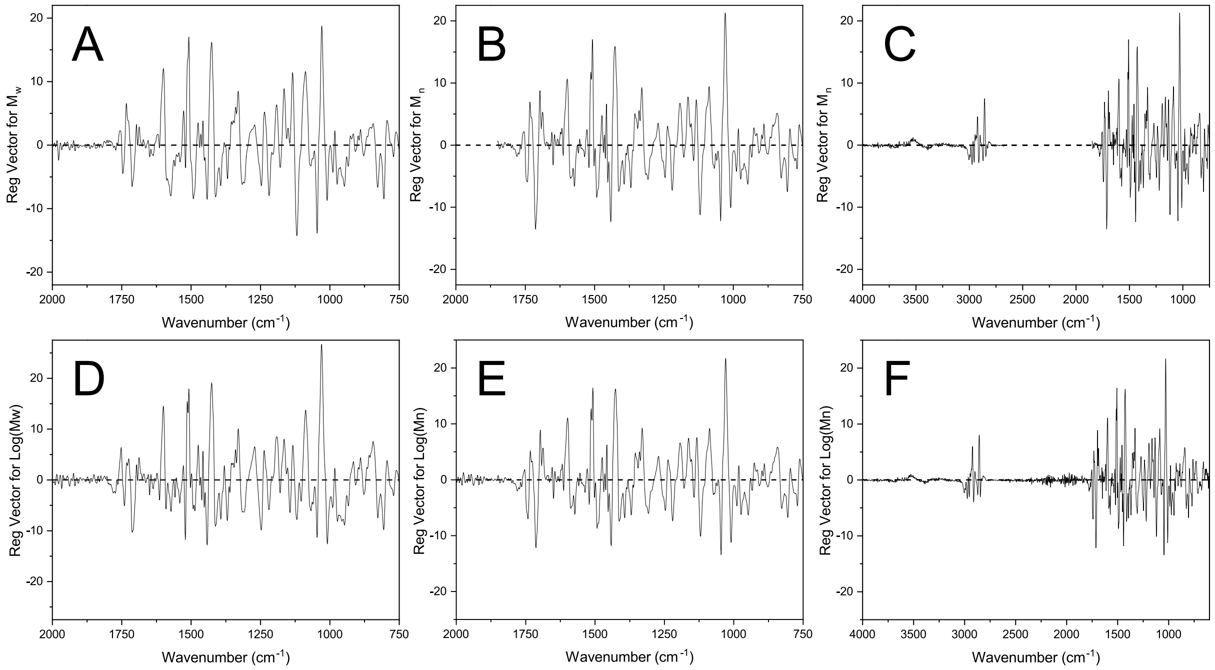

Figure S3. Full regression vectors for PLS models MW characteristics a) M_w, c) M_n, d) log(M_w), f) log(M_n), as measured by GPC and the predicted values as obtained from the ATR-IR spectra of the lignin oil samples. b) and e) show a zoom in of the regression vectors for M_n and log(M_n), respectively. 57 samples were utilised in total and were split into a Cal set (43 samples) and a Val set (14 samples).
